# Hypoxia-driven angiogenesis in breast cancer mechanisms and therapeutic targets: a narrative review

**DOI:** 10.1097/MS9.0000000000003411

**Published:** 2025-05-20

**Authors:** Emmanuel Ifeanyi Obeagu

**Affiliations:** Department of Biomedical and Laboratory Science, Africa University, Zimbabwe

**Keywords:** angiogenesis, breast cancer, hypoxia, therapeutic targets, VEGF

## Abstract

Breast cancer remains a significant global health challenge, with hypoxia playing a crucial role in its progression. Hypoxia, defined as reduced oxygen availability, is a hallmark of solid tumors and particularly influences the tumor microenvironment in breast cancer. Under hypoxic conditions, tumors activate a variety of molecular responses that promote survival, including the stabilization of hypoxia-inducible factors (HIFs). These transcription factors regulate the expression of pro-angiogenic genes, such as vascular endothelial growth factor (VEGF), which drive angiogenesis and support tumor growth. However, the vasculature formed under hypoxic conditions is often dysfunctional, contributing to tumor progression, metastasis, and resistance to therapies. This review explores the mechanisms by which hypoxia drives angiogenesis in breast cancer, emphasizing the roles of HIFs, VEGF signaling, and metabolic reprogramming. Angiogenesis, a critical process for tumor survival and growth, is primarily mediated by the induction of VEGF under hypoxic conditions. VEGF acts on endothelial cells to promote blood vessel formation, ensuring the tumor receives sufficient oxygen and nutrients. However, the vessels formed are typically leaky and inefficient, exacerbating the hypoxic environment and perpetuating a cycle of tumor progression. The metabolic reprogramming that occurs in hypoxic tumor cells, such as the shift toward glycolysis (the Warburg effect), also plays a pivotal role in sustaining angiogenesis. The resulting acidic conditions further enhance VEGF production and endothelial cell migration, supporting continued tumor growth and metastasis.

## Introduction

Breast cancer remains a leading cause of cancer-related mortality and morbidity worldwide. Despite significant advancements in early detection, surgical interventions, and systemic therapies, the prognosis for breast cancer patients remains largely influenced by factors within the tumor microenvironment (TME). One such factor that has garnered significant attention in recent years is hypoxia, a condition in which there is inadequate oxygen supply to tissues due to the imbalance between oxygen demand and delivery. Hypoxia is a hallmark feature of solid tumors, including breast cancer, and plays a critical role in tumor progression, metastasis, and resistance to therapies. The existence of hypoxic regions within the tumor contributes to cellular adaptations that allow cancer cells to survive and proliferate under harsh conditions^[1,2]^. The tumor microenvironment in breast cancer is often characterized by regions of low oxygen tension, arising as a result of rapid tumor growth that outpaces the formation of new blood vessels (angiogenesis). As the tumor grows and outstrips its vascular supply, the oxygen levels in the center of the tumor decrease, leading to hypoxia. The lack of oxygen creates an environment that drives tumor cells to initiate a series of molecular and cellular changes aimed at adapting to oxygen deprivation. These adaptations are mediated through the activation of hypoxia-inducible factors (HIFs), a family of transcription factors that regulate gene expression in response to low oxygen levels. The stabilization of HIFs in the hypoxic tumor environment triggers the expression of genes involved in cell survival, angiogenesis, metabolic reprogramming, immune evasion, and metastasis^[3,4]^. Among the most significant adaptive responses to hypoxia is the activation of angiogenesis, the process by which new blood vessels are formed to restore oxygen and nutrient supply to the tumor. Angiogenesis is primarily regulated by the upregulation of vascular endothelial growth factor (VEGF), a key pro-angiogenic factor that is activated by HIFs. VEGF promotes the proliferation, migration, and organization of endothelial cells into new blood vessels, ensuring that the growing tumor has a sufficient blood supply. However, despite the essential role of angiogenesis in maintaining tumor growth, the newly formed vasculature in hypoxic tumors is often abnormal, leaky, and functionally inefficient. This leads to further tumor hypoxia, creating a vicious cycle that fuels tumor progression and metastasis^[5,6]^.

**HIGHLIGHTS**

- Hypoxia triggers angiogenesis in breast cancer, facilitating tumor growth and metastasis.
- HIFs regulate critical angiogenic factors like VEGF, driving vascular development in tumors.
- Tumor microenvironment under hypoxic stress promotes therapeutic resistance.
- Targeting HIFs and VEGF can disrupt hypoxia-induced angiogenesis.
- Combination therapies targeting hypoxia and angiogenesis improve treatment efficacy and reduce metastasis.

In addition to promoting angiogenesis, hypoxia also leads to metabolic reprogramming in breast cancer cells. Under normal oxygen conditions, cells primarily rely on oxidative phosphorylation for energy production. However, in hypoxic environments, breast cancer cells shift toward glycolysis to meet their energy demands, even in the presence of oxygen. This shift, known as the Warburg effect, allows cancer cells to generate ATP rapidly through glycolysis, despite inefficient energy production. The metabolic shift also leads to the accumulation of lactate, which acidifies the tumor microenvironment. The acidic conditions further promote tumor cell invasiveness and immune evasion, enabling the tumor to progress and metastasize^[7,8]^. Hypoxia in the TME also plays a profound role in modulating the immune landscape of breast cancer. Oxygen deprivation suppresses the activity of cytotoxic immune cells, such as T lymphocytes and natural killer cells, which are essential for eliminating tumor cells. At the same time, hypoxia promotes the recruitment of immunosuppressive cells, including regulatory T cells (Tregs) and myeloid-derived suppressor cells (MDSCs), which dampen the immune response against the tumor. Moreover, hypoxia induces the expression of immune checkpoint molecules like PD-L1 on tumor cells, further impairing the ability of the immune system to target and destroy cancer cells. These immune-modulating effects of hypoxia create a microenvironment that supports tumor survival and progression^[9,10]^. In addition to immune modulation, hypoxia contributes to the phenotypic transformation of breast cancer cells through a process known as epithelial-to-mesenchymal transition (EMT). EMT is characterized by the loss of epithelial markers and the gain of mesenchymal features, which enhance the migratory and invasive potential of cancer cells. Hypoxia-induced EMT is driven by the upregulation of key transcription factors such as Snail, Twist, and Zeb1, which suppress the expression of epithelial adhesion molecules like E-cadherin while promoting the expression of mesenchymal markers like N-cadherin and vimentin. The induction of EMT enables breast cancer cells to detach from the primary tumor, invade surrounding tissues, and ultimately metastasize to distant organs, contributing to disease progression and poor clinical outcomes^[11,12]^. One of the most challenging consequences of hypoxia in breast cancer is the development of therapeutic resistance. Both chemotherapy and radiation therapy rely on the generation of reactive oxygen species (ROS) to induce DNA damage and kill cancer cells. However, in hypoxic tumors, the lack of oxygen limits ROS production, thereby reducing the efficacy of these therapies. Moreover, hypoxic regions within tumors often contain a subpopulation of dormant or quiescent cancer cells, which are less susceptible to the cytotoxic effects of chemotherapy. These therapy-resistant cells can lead to tumor recurrence and metastasis, further complicating treatment strategies and patient outcomes^[13,14]^.

## Aim

The aim of this review is to explore the mechanisms underlying hypoxia-driven angiogenesis in breast cancer, examining how oxygen deprivation within the tumor microenvironment promotes the formation of abnormal blood vessels that contribute to tumor growth, metastasis, and therapeutic resistance.

## Justification of the review

Hypoxia is a critical feature of the tumor microenvironment in breast cancer, and its role in promoting angiogenesis is well-established. Despite advancements in breast cancer diagnosis and treatment, the development of resistance to therapy and tumor metastasis remain major challenges that contribute to the high morbidity and mortality associated with the disease. Hypoxia-driven angiogenesis, which facilitates tumor growth and metastasis by providing an inadequate but functional blood supply, is a key mechanism behind these challenges. Current therapies targeting angiogenesis have shown limited success in clinical settings, primarily due to the complexity of tumor vasculature and the compensatory mechanisms that can be activated in response to angiogenesis inhibition. Therefore, a comprehensive review that highlights the intricate relationship between hypoxia, angiogenesis, and tumor progression in breast cancer is needed. Additionally, exploring the therapeutic strategies that aim to target hypoxia-driven angiogenesis will contribute to the identification of new avenues for treatment that can complement existing therapies^[8,15]^. This review will provide a platform for the integration of basic research findings with clinical applications, thereby accelerating the development of more effective and targeted therapeutic interventions for breast cancer.

## Review methods

### Literature search strategy

A systematic search of relevant scientific databases, including PubMed, Scopus, and Google Scholar, was conducted. The search terms used included “hypoxia,” “angiogenesis,” “breast cancer,” “vascular endothelial growth factor (VEGF),” “hypoxia-inducible factors (HIFs),” and “therapeutic targets.” These terms were combined with Boolean operators to ensure the inclusion of articles that provided comprehensive data on hypoxia-driven angiogenesis in breast cancer and potential therapeutic strategies targeting these pathways.

### Inclusion and exclusion criteria

The review focused on peer-reviewed articles, clinical studies, preclinical studies, and review articles. Studies included in the review were those that addressed the following aspects:

- Mechanisms of hypoxia-driven angiogenesis in breast cancer.
- Role of HIFs, VEGF, and other molecular signaling pathways in angiogenesis.
- Preclinical and clinical studies targeting hypoxia-driven angiogenesis in breast cancer.
- Mechanisms of resistance to angiogenesis inhibitors.
- Outcomes of combination therapies aimed at hypoxia and angiogenesis inhibition.

Excluded studies included those that focused on non-breast cancer types, animal studies that lacked relevance to human breast cancer, and studies that did not provide molecular mechanistic insights or clinical data.

## Mechanisms of hypoxia-driven angiogenesis in breast cancer

Hypoxia is a defining feature of many solid tumors, including breast cancer, where it plays a central role in tumor progression. As tumors grow, their demand for oxygen often outpaces the ability of existing blood vessels to supply oxygen, leading to hypoxic regions within the tumor. Hypoxia-driven angiogenesis is a crucial process that helps tumors restore oxygen and nutrient supply by inducing the formation of new blood vessels. This mechanism is regulated by a complex interplay of molecular signals, primarily orchestrated by HIFs, which are key transcription factors activated under low oxygen conditions[16].

### Hypoxia-inducible factor (HIF) activation

The central regulator of hypoxia-driven angiogenesis is the family of HIFs, particularly HIF-1α. Under normal oxygen conditions, HIF-1α is rapidly degraded through the ubiquitin-proteasome pathway. However, in hypoxic environments, the degradation pathway is inhibited, leading to the stabilization and accumulation of HIF-1α in the cell. Once stabilized, HIF-1α translocates to the nucleus, where it forms a heterodimer with HIF-1β. This dimerization allows the transcriptional activation of a variety of genes involved in the hypoxic response, including those that regulate angiogenesis. Among the most important of these genes is VEGF, a potent pro-angiogenic factor that plays a pivotal role in tumor vasculature formation^[17,18]^.

### VEGF and angiogenesis

VEGF is one of the primary mediators of hypoxia-induced angiogenesis. Under hypoxic conditions, the binding of HIF-1α to the promoter region of the VEGF gene induces its expression. VEGF then interacts with VEGF receptors on endothelial cells, triggering a cascade of signaling events that promote endothelial cell proliferation, migration, and tube formation. This leads to the formation of new blood vessels, which are critical for providing tumors with the necessary oxygen and nutrients for continued growth. However, in the context of breast cancer, these newly formed blood vessels are often structurally abnormal and dysfunctional, with irregular branching and leaky endothelial junctions. As a result, the vasculature fails to effectively deliver oxygen to the tumor, perpetuating a cycle of hypoxia and angiogenesis^[19,20]^.

### Other pro-angiogenic factors and signaling pathways

In addition to VEGF, hypoxia also induces the expression of other pro-angiogenic factors, such as fibroblast growth factors (FGFs), angiopoietins, and platelet-derived growth factors (PDGFs), which contribute to angiogenesis in the tumor microenvironment. These factors act through specific receptors on endothelial cells, leading to the activation of multiple intracellular signaling pathways that promote cell survival, proliferation, and migration. For instance, FGFs activate the mitogen-activated protein kinase (MAPK) and phosphoinositide 3-kinase (PI3K)/Akt pathways, which are critical for endothelial cell proliferation and survival. Angiopoietins, particularly angiopoietin-2 (Ang-2), contribute to the destabilization of the existing vasculature, making it more susceptible to the effects of VEGF-induced angiogenesis. Collectively, these factors work synergistically to ensure the efficient formation of new blood vessels within the tumor^[21,22]^.

### Hypoxia-induced matrix remodeling and tumor stiffness

Hypoxia also affects the extracellular matrix (ECM), which plays a critical role in angiogenesis and tumor progression. The tumor microenvironment is often characterized by ECM remodeling, which is regulated by hypoxia through the induction of matrix metalloproteinases (MMPs). These enzymes degrade ECM components, such as collagen and fibronectin, facilitating the migration and invasion of endothelial cells during angiogenesis. Additionally, hypoxia-induced changes in ECM composition and mechanical properties, such as increased stiffness, can alter the behavior of endothelial cells and other stromal cells within the tumor. Tumor-associated macrophages (TAMs), for instance, are recruited by hypoxic signals and contribute to ECM remodeling, further promoting angiogenesis. This interplay between hypoxia, ECM remodeling, and angiogenesis enhances tumor growth and facilitates metastasis by creating a more permissive environment for tumor cell migration and invasion^[23,24]^.

### Role of notch signaling in angiogenesis

The Notch signaling pathway is another key regulator of angiogenesis that is activated by hypoxia. Notch signaling plays a crucial role in maintaining the balance between endothelial cell proliferation and differentiation during angiogenesis. In response to hypoxic signals, the activation of Notch receptors on endothelial cells leads to the transcription of genes that promote the stabilization of new blood vessels and prevent excessive sprouting. Notch signaling also regulates the expression of vascular endothelial cadherin, a cell adhesion molecule that helps maintain endothelial cell-cell junctions and the integrity of the vasculature. However, aberrant Notch signaling, often induced by hypoxia, can lead to excessive angiogenesis and the formation of dysfunctional blood vessels that contribute to tumor progression and metastasis^[25,26]^.

### Hypoxia-driven angiogenesis and metastasis

Hypoxia-driven angiogenesis is not only crucial for tumor growth but also plays a significant role in breast cancer metastasis. The formation of new blood vessels provides an avenue for tumor cells to enter the bloodstream and spread to distant organs. Additionally, the leaky and poorly organized vasculature in hypoxic tumors facilitates the extravasation of tumor cells into surrounding tissues, where they can establish secondary tumors. Hypoxia-induced angiogenesis also creates a pro-inflammatory microenvironment that promotes tumor cell survival and metastatic potential. TAMs and other immune cells contribute to this process by secreting cytokines and chemokines that further support angiogenesis and metastasis^[27,28]^.

### Implications for targeted therapies

Given the critical role of hypoxia-driven angiogenesis in breast cancer progression, targeting the pathways involved in this process has become a promising therapeutic strategy. Anti-angiogenic therapies, such as bevacizumab (a monoclonal antibody targeting VEGF), have shown some success in clinical trials by inhibiting VEGF signaling and reducing tumor vasculature. However, these therapies have had limited success in the long term due to the development of resistance and the heterogeneous nature of tumors. To overcome these challenges, strategies targeting multiple pro-angiogenic pathways, including VEGF, FGF, and Notch signaling, are being explored. Additionally, hypoxia-activated prodrugs (HAPs) that selectively target hypoxic regions within tumors are being developed to improve therapeutic efficacy and minimize toxicity to normal tissues^[29,30]^.

### Combination strategies with chemotherapy and immunotherapy

**Figure 1.**
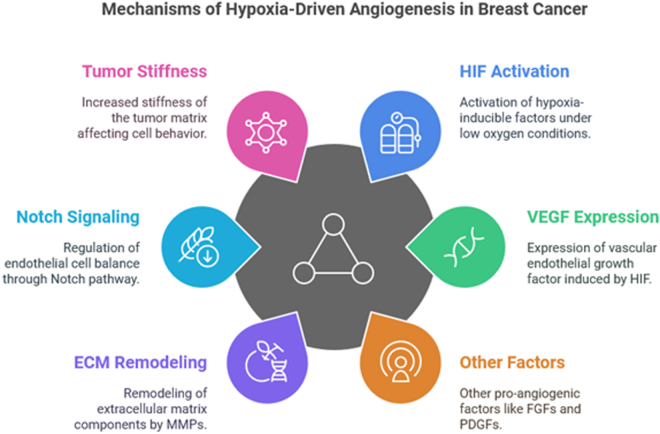
Mechanisms of hypoxia-driven angiogenesis in breast cancer.

The combination of anti-angiogenic therapies with chemotherapy and immunotherapy is an emerging strategy aimed at improving treatment outcomes in breast cancer. By normalizing tumor vasculature, anti-angiogenic agents can enhance the delivery of chemotherapy drugs to tumor cells and improve their effectiveness. Similarly, targeting hypoxia-induced immune suppression and enhancing anti-tumor immune responses through immunotherapy holds promise for reversing the immune evasion caused by hypoxic conditions. Combination therapies are being actively investigated in clinical trials, and early results suggest that these strategies may significantly improve clinical outcomes in patients with breast cancer (Fig. 1)^[31,32]^.

## Therapeutic targeting of hypoxia-driven angiogenesis in breast cancer

Hypoxia-driven angiogenesis has emerged as a critical process in the progression of breast cancer, contributing to tumor growth, metastasis, and therapeutic resistance. Given the central role of hypoxia in facilitating tumor vascularization, therapeutic strategies targeting hypoxia-driven angiogenesis have become a focal point in breast cancer treatment. These therapies aim to block the formation of new blood vessels within tumors, thereby limiting the tumor’s ability to grow and metastasize. This section reviews current and emerging therapeutic approaches targeting hypoxia-driven angiogenesis in breast cancer[33].

### Anti-angiogenic therapies: targeting VEGF and its receptors

The most widely studied therapeutic approach for targeting hypoxia-driven angiogenesis in breast cancer is the inhibition of VEGF and its receptors. VEGF is a key mediator of angiogenesis, and its expression is strongly induced by hypoxic conditions. VEGF binds to its receptors on endothelial cells, triggering pathways that promote angiogenesis. Bevacizumab, a monoclonal antibody that targets VEGF-A, has been developed to block VEGF binding to its receptors, thereby inhibiting angiogenesis. Clinical trials have shown that bevacizumab can effectively reduce tumor vasculature and improve progression-free survival in patients with breast cancer. However, the benefits of bevacizumab are often limited, and resistance mechanisms, such as the upregulation of alternative pro-angiogenic factors like FGF, can diminish its effectiveness. These challenges highlight the need for more comprehensive approaches targeting multiple angiogenic pathways^[34,35]^.

### Inhibition of FGF and other pro-angiogenic factors

In addition to VEGF, other pro-angiogenic factors, such as FGFs, PDGFs, and angiopoietins, play a significant role in the regulation of angiogenesis in the hypoxic tumor microenvironment. FGFs, for instance, activate the MAPK and PI3K/Akt pathways, which are essential for endothelial cell survival and proliferation. Inhibiting these pathways has been shown to impair angiogenesis and tumor growth. Several small-molecule inhibitors targeting FGF receptors (FGFRs) are under investigation in clinical trials for breast cancer. By blocking FGFR signaling, these inhibitors can reduce endothelial cell proliferation and disrupt angiogenesis. Similarly, targeting the angiopoietin-Tie2 signaling axis has shown promise in preclinical models, as angiopoietins contribute to the destabilization of the vasculature and promote aberrant angiogenesis. Combination strategies that target VEGF and other angiogenic factors, such as FGF, are being explored to overcome resistance and improve treatment efficacy^[36,37]^.

### Hypoxia-activated prodrugs (HAPs)

Hypoxia-activated prodrugs (HAPs) represent an innovative therapeutic approach that exploits the unique characteristics of hypoxic tumor regions. These prodrugs are designed to remain inert in oxygenated environments but become activated under hypoxic conditions, selectively delivering cytotoxic agents to the hypoxic regions of tumors. One of the most well-known HAPs is TH-302, which undergoes bioreductive activation in low-oxygen environments, leading to the release of a toxic drug that selectively targets hypoxic tumor cells. In addition to their cytotoxic effects on tumor cells, HAPs may also help disrupt the pro-angiogenic signaling pathways activated by hypoxia. By selectively targeting hypoxic regions, HAPs can reduce the oxygen supply to the tumor and potentially enhance the effectiveness of other therapies, such as chemotherapy and radiation therapy[38].

### Targeting the hypoxia-inducible factor (HIF) pathway

The HIF pathway is a central regulator of hypoxia-driven angiogenesis. Under normal oxygen conditions, HIF-1α is rapidly degraded; however, under hypoxic conditions, HIF-1α is stabilized and activates the transcription of genes involved in angiogenesis, including VEGF. Targeting HIF-1α has therefore become a promising therapeutic strategy. Several small molecules and inhibitors have been developed to block HIF-1α stabilization and activity. For example, drugs such as digoxin and PX-478 inhibit HIF-1α expression by interfering with the protein degradation machinery or the interaction between HIF-1α and its co-activators. By blocking the HIF pathway, these agents reduce the expression of pro-angiogenic factors like VEGF, thereby limiting angiogenesis and tumor progression. Although HIF inhibitors have shown promise in preclinical studies, their clinical development has been hindered by concerns regarding potential off-target effects and the complexity of the hypoxia response^[39,40]^.

### Combination therapies

Combination therapies that integrate hypoxia-targeting agents with other treatment modalities are an area of active research in breast cancer therapy. One promising strategy involves combining anti-angiogenic therapies with immunotherapies. Hypoxia-induced immune suppression, particularly the recruitment of immunosuppressive cells like Tregs and MDSCs, impedes the effectiveness of immune-based therapies. By normalizing the vasculature and reducing hypoxia through anti-angiogenic agents, tumors may become more accessible to immune cells, enhancing the effectiveness of immunotherapies. In preclinical models, the combination of anti-VEGF agents with immune checkpoint inhibitors, such as anti-PD-1 or anti-CTLA-4 antibodies, has shown promising results in improving anti-tumor immunity. The ability to reverse hypoxia-induced immune suppression while simultaneously targeting angiogenesis holds significant potential for improving the treatment of breast cancer^[41,42]^.

### Targeting tumor microenvironment and stromal components

While much attention has been focused on targeting endothelial cells and angiogenesis, the TME plays a crucial role in regulating hypoxia-driven angiogenesis. The stroma, including fibroblasts, immune cells, and ECM components, significantly influences tumor progression and response to treatment. Hypoxia induces changes in the TME, leading to the secretion of pro-angiogenic factors and ECM remodeling. Targeting stromal components, such as cancer-associated fibroblasts (CAFs) and macrophages, may help modulate the hypoxic response and disrupt angiogenesis. For instance, CAFs secrete factors like TGF-β, which can promote the formation of an abnormal vasculature. Targeting these stromal cells with specific inhibitors could disrupt the pro-angiogenic signals within the TME, enhancing the efficacy of anti-angiogenic therapies and other cancer treatments^[43-46]^.

### Nanomedicine approaches for targeting hypoxia and angiogenesis

Nanomedicine offers a promising avenue for delivering hypoxia-targeting therapies with high precision and reduced toxicity. Nanoparticles can be engineered to carry drugs that specifically target hypoxic regions, thereby improving drug delivery and bioavailability. For example, nanoparticles can be functionalized with targeting ligands that recognize receptors overexpressed in hypoxic tumor regions, such as integrins or specific endothelial markers. These targeted nanoparticles can deliver anti-angiogenic agents, chemotherapy drugs, or gene therapies directly to hypoxic tissues, minimizing exposure to healthy tissues. Moreover, nanoparticles can be designed to respond to the low-oxygen environment, activating or releasing their payload selectively within hypoxic regions. This approach offers the potential to improve the specificity and efficacy of hypoxia-targeted therapies while reducing systemic side effects^[47-52]^.

### Overcoming resistance to anti-angiogenic therapies

**Figure 2.**
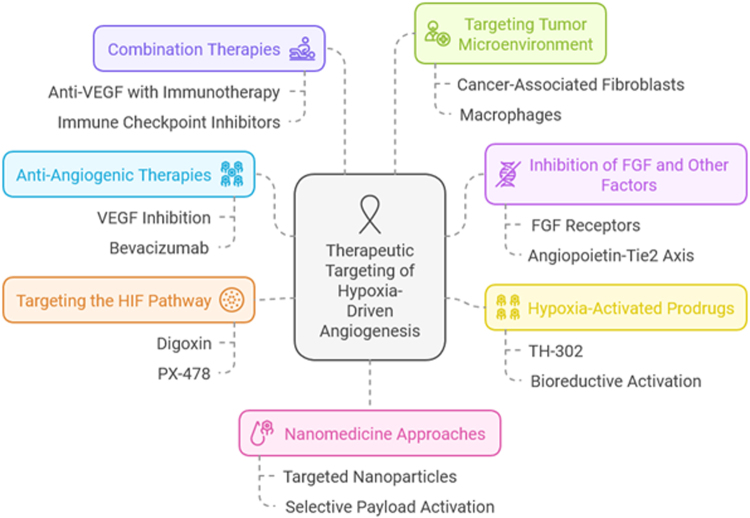
Therapeutic targeting of hypoxia-driven angiogenesis in breast cancer.

One of the major challenges in targeting hypoxia-driven angiogenesis in breast cancer is the development of resistance to anti-angiogenic therapies. Resistance mechanisms can occur through several pathways, including the upregulation of alternative pro-angiogenic factors, such as FGF, or the activation of alternative endothelial cell signaling pathways. In addition, tumor cells can adapt to reduced oxygen levels by switching to alternative metabolic pathways that do not rely on angiogenesis for survival. To overcome resistance, combination therapies that target multiple angiogenic pathways or combine anti-angiogenic agents with chemotherapy, radiation, or immune checkpoint inhibitors are being explored. Identifying biomarkers of resistance and developing personalized treatment strategies are crucial for optimizing the clinical success of hypoxia-targeting therapies (Fig. 2)^[53-55]^.

## Recommendations for targeting hypoxia-driven angiogenesis in breast cancer

Breast cancer remains one of the leading causes of cancer-related mortality, with angiogenesis playing a central role in tumor progression and metastasis. Tumors, including breast cancer, often experience hypoxia due to rapid growth outpacing their vascular supply. This hypoxic microenvironment is a key driver of angiogenesis, providing essential nutrients and oxygen for continued tumor growth. Hypoxia-induced angiogenesis is primarily regulated by HIFs, which, through the activation of pro-angiogenic factors like VEGF, promote new blood vessel formation. Targeting this process offers significant potential for therapeutic intervention.

### Anti-VEGF monoclonal antibodies

The use of anti-VEGF monoclonal antibodies, such as bevacizumab, to inhibit VEGF signaling and reduce angiogenesis in breast cancer. Anti-VEGF therapies, including bevacizumab, have shown promise in inhibiting angiogenesis in various cancers, including breast cancer. Bevacizumab works by binding to VEGF and preventing it from activating its receptors on endothelial cells, thus disrupting the formation of new blood vessels. Several clinical trials have demonstrated that bevacizumab, when used in combination with chemotherapy, can prolong progression-free survival in metastatic breast cancer. However, its impact on overall survival has been less conclusive, and resistance mechanisms, such as upregulation of alternative angiogenic pathways, may limit its long-term effectiveness. The level of evidence for this recommendation is moderate due to the mixed clinical outcomes observed in large-scale trials. Anti-VEGF therapies should be considered as part of combination treatments, but careful patient selection is essential to optimize therapeutic outcomes.

### HIF-1α inhibitors

Development and use of small molecule inhibitors targeting HIF-1α to block the transcriptional activity responsible for angiogenic gene expression in the hypoxic tumor microenvironment. HIF-1α is a key transcription factor that regulates several genes involved in angiogenesis, including VEGF. Inhibiting HIF-1α represents a direct strategy to block hypoxia-induced angiogenesis. Several small molecule inhibitors, such as digoxin and PX-478, have shown potential in preclinical models of breast cancer by reducing HIF-1α stabilization and decreasing angiogenesis. Early-phase clinical trials have also demonstrated the feasibility of targeting HIF-1α, though the full clinical impact is still being explored. The evidence supporting this strategy is primarily preclinical, with a growing body of early-phase clinical data suggesting promise. Continue to explore HIF-1α inhibitors in clinical trials, particularly in combination with existing therapies, to assess their potential in enhancing treatment efficacy and overcoming resistance.

### Targeting the extracellular matrix (ECM) and matrix metalloproteinases (MMPs)

Inhibition of matrix metalloproteinases (MMPs) to prevent ECM remodeling and endothelial cell migration, which are crucial steps in angiogenesis. The ECM plays a significant role in tumor progression and angiogenesis. Hypoxia-induced ECM remodeling facilitates endothelial cell migration and the formation of new blood vessels. MMPs, which degrade the ECM, are upregulated under hypoxic conditions and contribute to angiogenesis in breast cancer. Inhibiting MMPs has been shown to reduce angiogenesis and tumor growth in preclinical models of breast cancer. Clinical trials using broad-spectrum MMP inhibitors, however, have had mixed results, and concerns regarding the long-term safety and efficacy of MMP inhibition remain. Despite these challenges, targeting ECM remodeling remains a viable strategy with moderate supporting evidence. Further refinement of MMP inhibitors, focusing on selective targeting of specific MMPs involved in angiogenesis, is needed to improve therapeutic outcomes in breast cancer.

### Targeting angiopoietins and tie-2 receptor pathway

Development of therapies targeting the angiopoietin-Tie-2 receptor pathway to modulate blood vessel maturation and stability, which are dysregulated in breast cancer. The angiopoietin-Tie-2 receptor signaling pathway is critical in regulating endothelial cell function and vascular remodeling. Angiopoietins (Ang-1 and Ang-2) are involved in the maturation and stability of blood vessels. Under hypoxic conditions, Ang-2 is often upregulated, contributing to dysfunctional, leaky vessels, which are characteristic of tumors. Targeting this pathway by inhibiting Ang-2 or activating Ang-1 could restore proper vessel maturation and reduce tumor-associated angiogenesis. While promising preclinical data support this approach, clinical trials investigating angiopoietin antagonists in breast cancer are still in the early stages, and thus the evidence level remains low to moderate. Continue exploring the angiopoietin-Tie-2 axis as a potential therapeutic target, with a focus on designing selective inhibitors or modulators that can enhance vascular stability without affecting normal angiogenesis.

### Combination therapies: targeting multiple angiogenic pathways

Implement combination therapies targeting multiple angiogenic pathways (e.g., VEGF, HIF-1α, MMPs, and angiopoietins) to overcome compensatory mechanisms and improve the efficacy of anti-angiogenic treatments. The complexity of tumor angiogenesis, with its redundant signaling pathways and compensatory mechanisms, makes single-target therapies less effective in the long term. Combining anti-VEGF therapies with HIF inhibitors or MMP inhibitors may overcome resistance and improve overall treatment efficacy. Clinical trials have shown that combination therapies can provide more robust and durable responses in some cancer types. For example, combining bevacizumab with chemotherapy or radiotherapy has demonstrated improved progression-free survival in breast cancer, though the benefit in terms of overall survival remains uncertain. The evidence for this approach is moderate, as combination therapies are already in clinical use but still require optimization. Further investigation into combination therapies targeting multiple angiogenic pathways is crucial for enhancing treatment efficacy, and clinical trials should focus on identifying the best combinations for breast cancer patients.

### Personalized medicine: biomarker-guided therapy

Utilize biomarkers, such as HIF-1α, VEGF, and MMP levels, to guide the selection of anti-angiogenic therapies tailored to individual patients. Biomarkers can help identify patients most likely to benefit from anti-angiogenic treatments by predicting the level of hypoxia and angiogenic activity in tumors. For example, high VEGF expression or elevated HIF-1α levels in breast cancer tissue may indicate a higher likelihood of response to anti-VEGF therapies or HIF inhibitors. Personalized approaches to angiogenesis inhibition are gaining traction as a means of optimizing treatment regimens. However, while promising, the full implementation of biomarker-guided therapy in clinical practice requires further validation in prospective studies. Incorporate biomarkers into clinical practice to better tailor anti-angiogenic therapies and improve patient outcomes by matching treatments with the molecular characteristics of each tumor.

## Conclusion

Hypoxia-driven angiogenesis plays a pivotal role in the progression and aggressiveness of breast cancer. As tumors grow, they often outpace the development of a sufficient blood supply, creating hypoxic regions that foster molecular adaptations in cancer cells. These adaptations, primarily regulated by HIFs, promote key processes such as angiogenesis, metabolic reprogramming, immune evasion, and epithelial-to-mesenchymal transition, all of which contribute to tumor survival and metastasis. The resulting abnormal and dysfunctional vasculature further exacerbates the hypoxic condition, creating a vicious cycle that drives tumor progression and therapeutic resistance. The therapeutic targeting of hypoxia-driven angiogenesis offers significant promise for improving the treatment of breast cancer. Advances in anti-angiogenic therapies, hypoxia-activated prodrugs, and strategies aimed at modulating the tumor microenvironment provide potential avenues to disrupt the pro-tumorigenic adaptations of hypoxic cells. However, the complexity of hypoxia in breast cancer, including the development of resistance mechanisms and the immunosuppressive environment, presents substantial challenges. To address these challenges, a multi-targeted, personalized approach that combines hypoxia-targeting therapies with other treatment modalities, such as chemotherapy, immunotherapy, and radiation, is critical.

## Data Availability

Not applicable as this a narrative review.

## Research registration unique identifying number (UIN)

Not applicable as this a narrative review.
